# Sulfated Dextrans Enhance *In Vitro* Amplification of Bovine Spongiform Encephalopathy PrP^Sc^ and Enable Ultrasensitive Detection of Bovine PrP^Sc^

**DOI:** 10.1371/journal.pone.0013152

**Published:** 2010-10-04

**Authors:** Yuichi Murayama, Miyako Yoshioka, Kentaro Masujin, Hiroyuki Okada, Yoshifumi Iwamaru, Morikazu Imamura, Yuichi Matsuura, Shigeo Fukuda, Sadao Onoe, Takashi Yokoyama, Shirou Mohri

**Affiliations:** 1 Prion Disease Research Center, National Institute of Animal Health, Tsukuba, Japan; 2 Safety Research Team, National Institute of Animal Health, Tsukuba, Japan; 3 Hokkaido Animal Research Center, Shintoku, Japan; Rocky Mountain Laboratories, NIAID, NIH, United States of America

## Abstract

**Background:**

Prions, infectious agents associated with prion diseases such as Creutzfeldt-Jakob disease in humans, bovine spongiform encephalopathy (BSE) in cattle, and scrapie in sheep and goats, are primarily comprised of PrP^Sc^, a protease-resistant misfolded isoform of the cellular prion protein PrP^C^. Protein misfolding cyclic amplification (PMCA) is a highly sensitive technique used to detect minute amounts of scrapie PrP^Sc^. However, the current PMCA technique has been unsuccessful in achieving good amplification in cattle. The detailed distribution of PrP^Sc^ in BSE-affected cattle therefore remains unknown.

**Methodology/Principal Findings:**

We report here that PrP^Sc^ derived from BSE-affected cattle can be amplified ultra-efficiently by PMCA in the presence of sulfated dextran compounds. This method is capable of amplifying very small amounts of PrP^Sc^ from the saliva, palatine tonsils, lymph nodes, ileocecal region, and muscular tissues of BSE-affected cattle. Individual differences in the distribution of PrP^Sc^ in spleen and cerebrospinal fluid samples were observed in terminal-stage animals. However, the presence of PrP^Sc^ in blood was not substantiated in the BSE-affected cattle examined.

**Conclusions/Significance:**

The distribution of PrP^Sc^ is not restricted to the nervous system and can spread to peripheral tissues in the terminal disease stage. The finding that PrP^Sc^ could be amplified in the saliva of an asymptomatic animal suggests a potential usefulness of this technique for BSE diagnosis. This highly sensitive method also has other practical applications, including safety evaluation or safety assurance of products and byproducts manufactured from bovine source materials.

## Introduction

Prions, the infectious agents associated with transmissible spongiform encephalopathies such as scrapie in sheep, chronic wasting disease (CWD) in deer and elk, bovine spongiform encephalopathy (BSE), and Creutzfeldt-Jakob disease (CJD) in humans, are primarily comprised of PrP^Sc^, a protease-resistant misfolded isoform of the cellular prion protein PrP^C^ [1]. Prion diseases are fatal neurodegenerative disorders, and are characterized by the accumulation of PrP^Sc^ in the nervous tissues of infected subjects [2].

BSE is an emerging disease that first appeared in the United Kingdom in 1986 [3]. The cause of the BSE outbreak in the UK is believed to have been the result of feeding cattle meat and bone meal (MBM) contaminated with PrP^Sc^ acquired from rendering carcasses of BSE- or scrapie-infected ruminants [4], [5]. Since variant CJD (vCJD) is suspected to be attributable to infectious agents associated with BSE [6]–[8], prophylactic hygiene dictates that infected cattle be identified and eradicated.

Immunological methods such as enzyme-linked immunosorbent assays and western blotting (WB) have been widely used for the detection of PrP^Sc^. It is now possible to perform *in vitro* amplification of hamster scrapie PrP^Sc^ using the protein misfolding cyclic amplification (PMCA) technique [9]. Extremely small amounts of PrP^Sc^ can be detected by combining PMCA with WB [10], [11]. PMCA has been used to amplify PrP^Sc^ from mice [12], deer [13], sheep [14], and humans [15]. PMCA has also been applied to the detection of bovine PrP^Sc^ in cattle [16], and serial PMCA has been shown to improve detection sensitivity [17]. Although hyperefficient amplification of a mouse-adapted BSE strain has been demonstrated [18], there are no reports that cite ultrasensitive and direct detection of bovine PrP^Sc^ in cattle using the current PMCA method.

Since the concentration of PrP^Sc^ in tissues or body fluids of BSE-infected animals is expected to be extremely low, the development of a sensitive method for detecting PrP^Sc^ in infected cattle is important. In the present study, we developed an extremely efficient method that is suitable for bovine PrP^Sc^ amplification. The method, which involves amplifying BSE PrP^Sc^ in the presence of sulfated dextran compounds, enables sensitive detection of PrP^Sc^ at levels equivalent to those obtained for detection of hamster PrP^Sc^ [19]. This method is capable of amplifying very small amounts of PrP^Sc^ from the saliva, palatine tonsils, lymph nodes, ileocecal region, and muscular tissues of BSE-affected cattle. The technology will result in a marked improvement in BSE safety.

## Results

### Sulfated dextran compounds enhanced BSE PrP^Sc^ amplification

**Figure 1:**
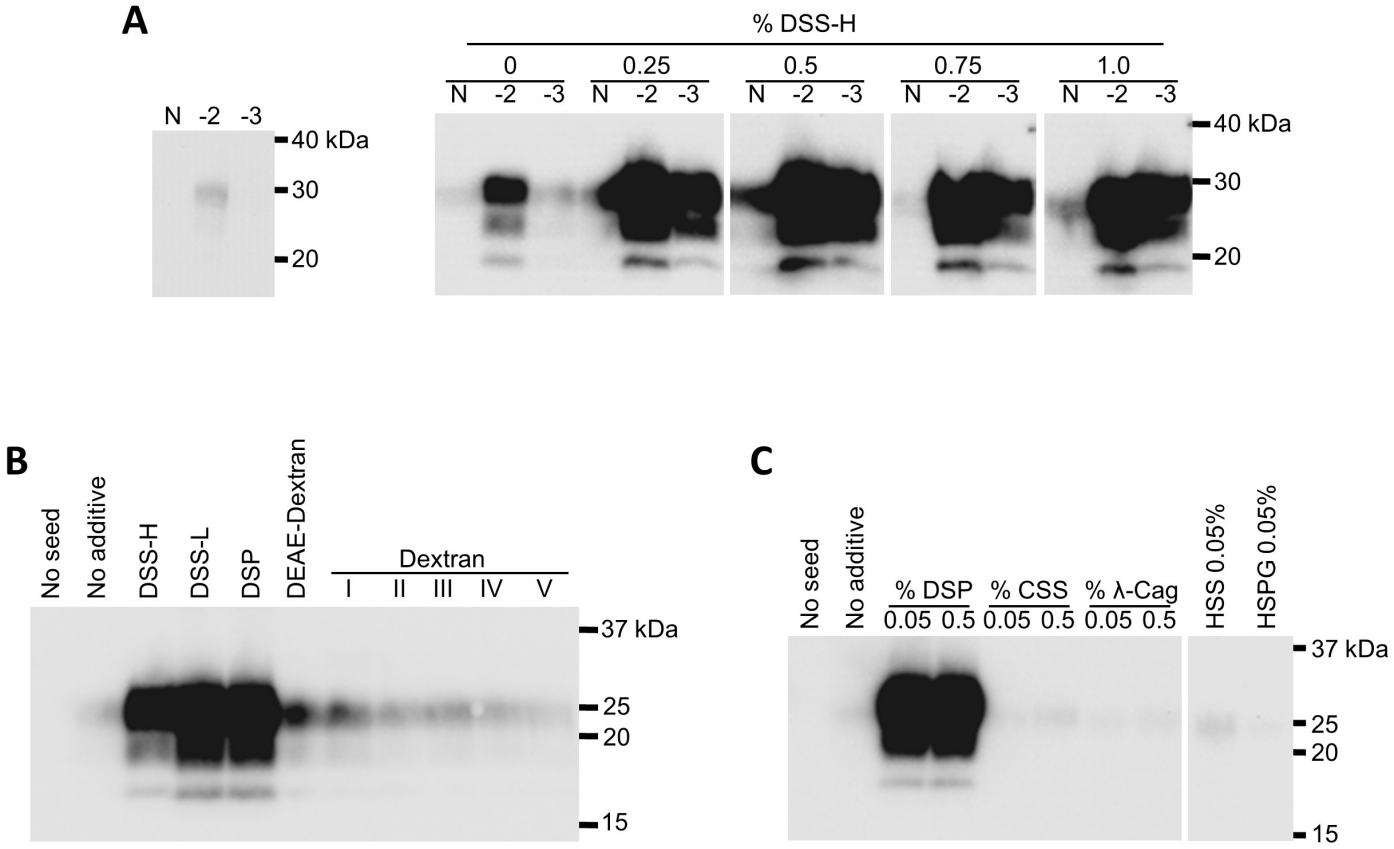
The effect of polysaccharides on the amplification of PrP^Sc^ derived from BSE-affected cattle. **A**. The effect of sodium dextran sulfate with a high molecular weight (MW) ranging from 900 to 2000 kDa (sodium dextran sulfate, DSS-H) on BSE-PrP^Sc^ amplification. The PrP^Sc^ seed (10% brain homogenate) was diluted to 10^−2^ to 10^−3^ in PrP^C^ substrate, and the diluted samples were amplified in the presence of DSS-H at 0–1%. The samples before (left panel) and after (right panel) amplification were analyzed by WB after digestion with PK. “N” designates controls in which the PrP^C^ substrate alone was treated in the same manner. **B**. The effect of dextran compounds on BSE-PrP^Sc^ amplification. The PrP^Sc^ seed was diluted to 10^−4^, and amplification was performed in the presence or absence (“No additive”) of dextran compounds at 0.5%. “No seed” designates the control in which the PrP^C^ substrate alone was amplified without dextran compounds. DSS-L: sodium dextran sulfate with a low MW, ranging from 5 to 6 kDa; DSP: potassium dextran sulfate (1.5 to 1.9 kDa); DEAE-dextran hydrochloride (50 kDa); dextran I (15–20 kDa), II (35–50 kDa), III (50–70 kDa), IV (200 kDa), and V (190–230 kDa). **C**. The effect of glycosaminoglycans (sodium chondroitin sulfate C: CSS; sodium heparan sulfate: HSS; heparan sulfate proteoglycan: HSPG) and a sulfated polysaccharide (λ-carrageenan: λ-Cag) on BSE-PrP^Sc^ amplification. The PrP^Sc^ seed was diluted to 10^−4^, and amplification was performed in the presence of each reagent at the final concentration indicated in the figure.

In general, the efficiency of BSE PrP^Sc^ amplification using PMCA was low compared to amplification of hamster and mouse scrapie PrP^Sc^ (Figure 1A). We attempted to improve BSE PrP^Sc^ amplification efficiency using high temperature conditions (39°C and higher). Various reagents were screened for their ability to prevent thermal denaturation of the brain homogenate, and we found that sulfated dextran compounds were suitable for this purpose. Unexpectedly, the BSE PrP^Sc^ amplification efficiency was greatly increased when amplification was performed in the presence of sulfated dextran compounds at 37°C.

The signal intensities of amplified PrP^Sc^ upon WB were significantly higher in samples containing sodium dextran sulfate with a high molecular weight of 900–2000 kDa (DSS-H) at a final concentration of 0.25–1% (Figure 1A, right panel). This enhancement was dependent upon the polarity and molecular size of the dextran compound. Smaller anionic sodium dextran sulfate (DSS-L) and potassium dextran sulfate (DSP) were more effective than the high molecular weight compounds, but positively charged DEAE-dextran and dextrans covering a range of molecular weights had little or no effect on amplification (Figure 1B). Glycosaminoglycans that are distributed throughout animal tissues and a sulfated polysaccharide were not effective at the concentrations examined (Figure 1C).

**Figure 2:**
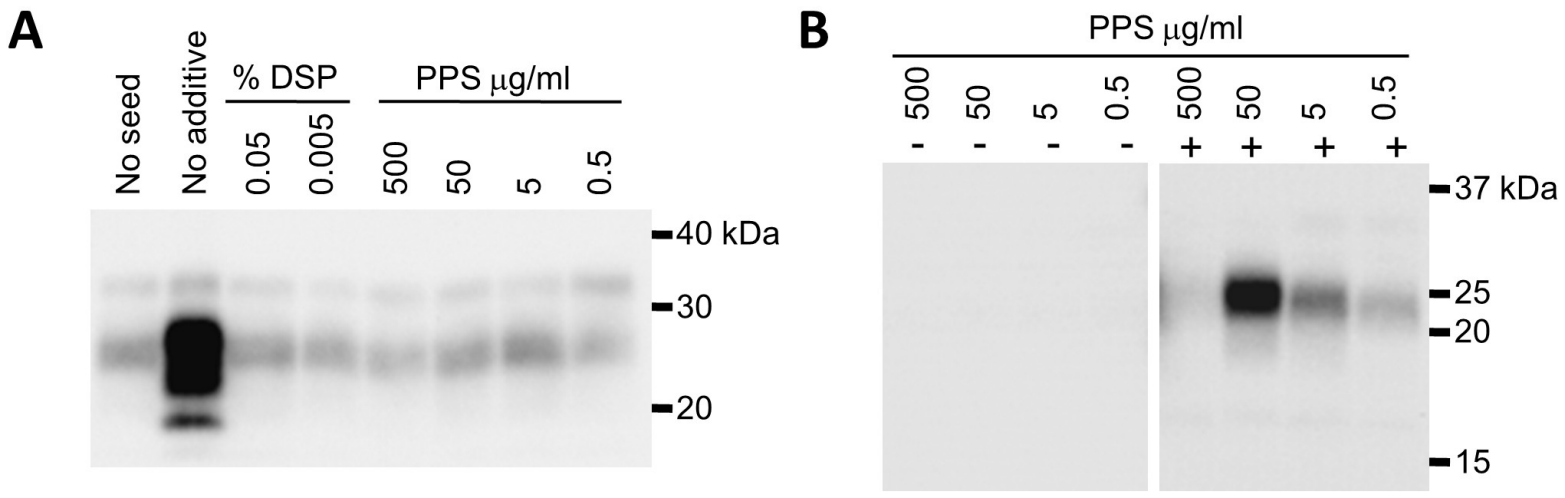
The effect of DSP and PPS on the amplification of scrapie and BSE PrP^Sc^. **A**. The PrP^Sc^ seed (10% brain homogenate from scrapie-infected sheep) was diluted to 10^−3^, and amplification was performed in the presence or absence (“No additive”) of sulfated compounds (potassium dextran sulfate: DSP and pentosan polysulfate: PPS). “No seed” designates the control in which PrP^C^ substrate (10% homogenate of normal mouse brain) was amplified without sulfated compounds. **B**. Negative symbols indicate the results obtained for the control samples without BSE PrP^Sc^ seed, while positive symbols indicate the results obtained for the samples containing BSE PrP^Sc^ seed diluted to 10^−4^. Amplification was performed in the presence of PPS at the concentrations indicated.

In contrast, *in vitro* amplification of sheep scrapie PrP^Sc^ was completely inhibited by the addition of DSP to the reaction mixture (Figure 2A). A similarly inconsistent experimental result was obtained with pentosan polysulfate (PPS), which may be effective in treating CJD [20], [21]. This reagent acted as an inhibitor of sheep scrapie PrP^Sc^ amplification (Figure 2A), but induced a low-level, dose-dependent amplification of BSE PrP^Sc^ (Figure 2B).

### Detection limit of BSE PrP^Sc^ using DSP-PMCA techniques

**Figure 3:**
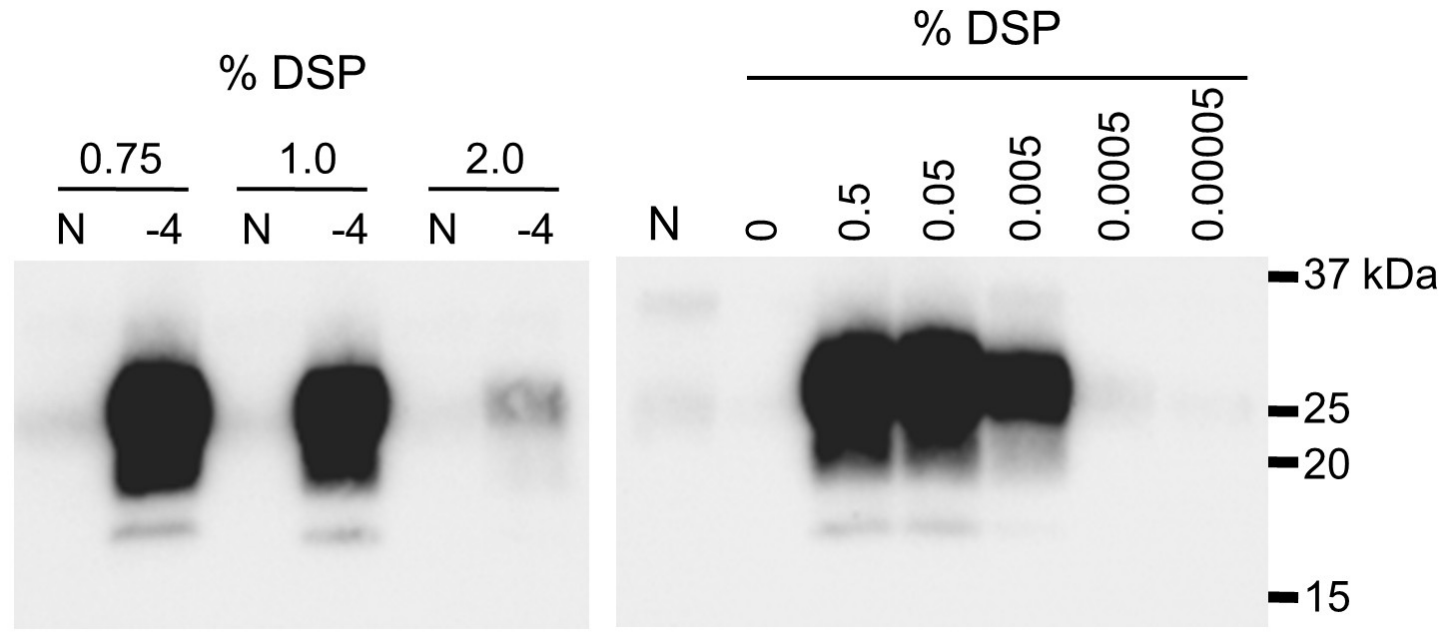
Optimal DSP concentration for BSE PrP^Sc^ amplification. The PrP^Sc^ seed was diluted to 10^−4^, and amplification was performed in the presence of the potassium dextran sulfate (DSP). “N” designates the control in which only PrP^C^ substrate was amplified.

**Figure 4:**
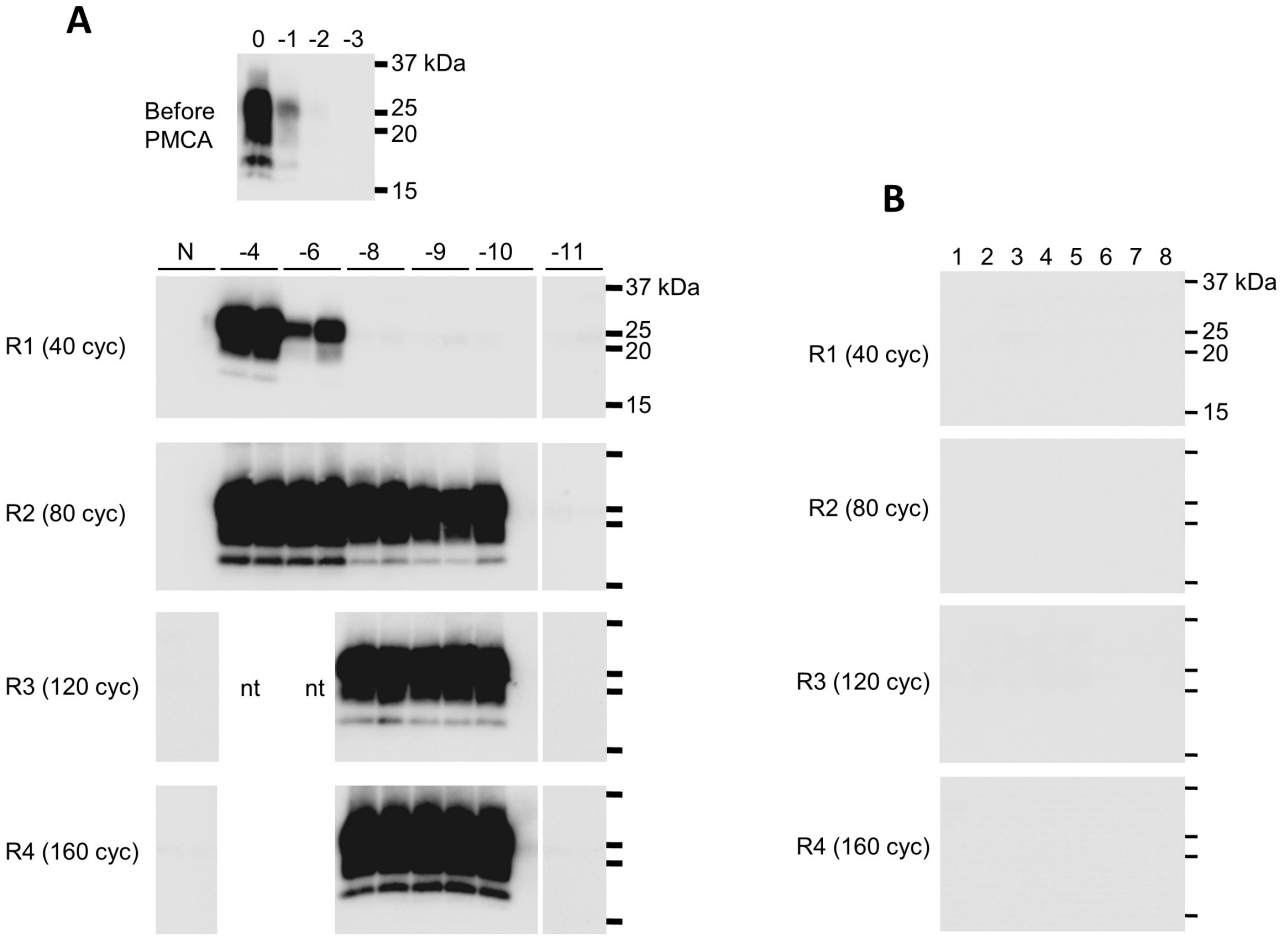
BSE-PrP^Sc^ detection sensitivity. **A**. The PrP^Sc^ seed was diluted to 10^−4^ to 10^−11^ with PrP^C^ substrate, and samples were serially amplified in the presence of 0.5% potassium dextran sulfate (DSP). The duplicate amplified samples were analyzed after each round of amplification (R1–R4) by WB after PK digestion. **B**. No spontaneous generation of PrP^Sc^ was observed. Samples labeled “1” to “8” contained only PrP^C^ substrate and were amplified in the presence of 0.5% DSP.

The optimal concentration of DSP was estimated to be in the range of 0.05–0.75% (Figure 3); therefore, we used 0.5% DSP for subsequent experiments. We determined the detection limit of the DSP-PMCA technique and confirmed that PrP^Sc^ present in a 10^−6^ dilution of infected brain homogenate could be detected after one round of amplification, and both 10^−8^ and 10^−9^ dilutions tested positive for PrP^Sc^ after two rounds of amplification (Figure 4A). A PrP^Sc^ signal was detected in one of the 10^−10^ dilution samples, but no signal was detected in the more extreme dilution range, even after four rounds of amplification. Thus, the PrP^Sc^ detection sensitivity was improved 10^8^ times compared to no amplification. The 50% lethal dose (LD50) per gram of brain homogenate used as seed was determined in a previous study [22] by a bioassay using Tg(BoPrP)4092HOZ/Prnp^0/0^ (TgBoPrP) transgenic mice [23]. The infectious titer was 10^6.7^ LD50/g, and infectivity was confirmed in mice intracerebrally inoculated with up to a 10^−4^ dilution of the 10% brain homogenate. Therefore, our improved method was 10^5^ times more sensitive than the bioassay. The generation of spontaneous PrP^Sc^, as has been reported for amplification in the presence of polyanions [24], [25], was not observed with four rounds of amplification (Figure 4B).

### Infectivity of the PMCA product

**Figure 5:**
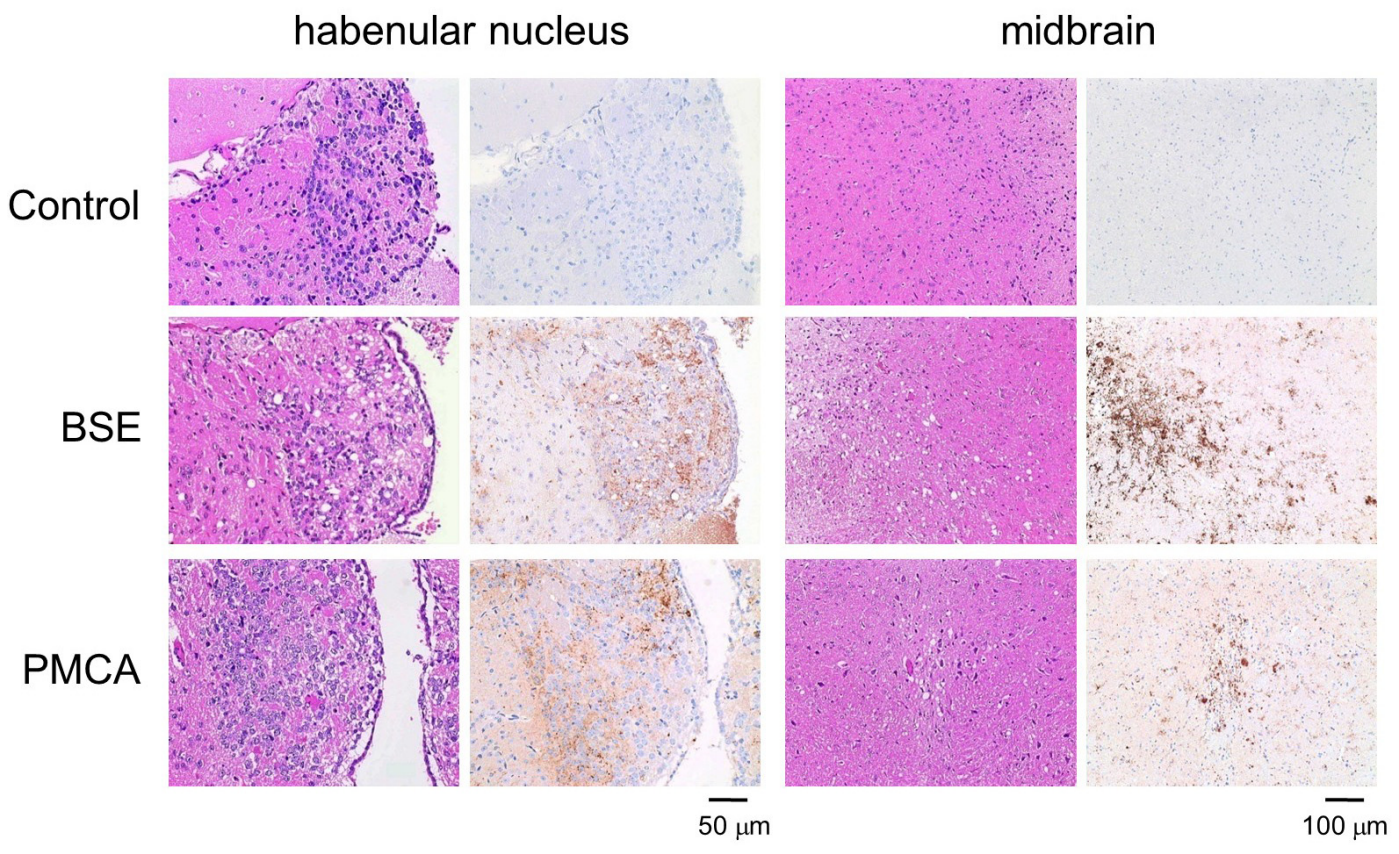
Immunohistological analysis of mice that succumbed to disease following intracerebral inoculation with PMCA product. Results from mice inoculated with a 10% brain homogenate from BSE-infected cattle are shown for comparison. No vacuolation or PrP^Sc^ accumulation was observed in the control mice inoculated with 0.05% DSP.

The PMCA product obtained after six rounds of amplification was diluted 10-fold and inoculated intracerebrally into TgBoPrP transgenic mice that overexpress bovine PrP^C^. Mice inoculated with the PMCA sample died after a mean of 243 days (Table 1). Control mice administered DSP (0.05%) or PrP^Sc^ (3.2×10^−12^ dilution) at concentrations corresponding to the BSE seed dilution in the PMCA sample survived more than 500 days. The results of immunohistochemical analysis of the habenular nuclei and the midbrains from control and treated mice are shown in Figure 5. Vacuolation and PrP^Sc^ accumulation, which was occasionally observed as plaque-like PrP^Sc^ aggregates, were found in mice inoculated with the PMCA sample or the BSE-affected cattle brain homogenate, indicating that the amplified PrP^Sc^ was infectious and caused lesions typical of prion diseases.

**Table 1:** Mean incubation time of TgBoPrP mice following intracerebral inoculation.

Inocula	Transmission rate (total death/total number)	Mean incubation time ± SD (days)
DSP dilution control (0.05%)	0% (0/3)	>532
BSE PrP^Sc^ dilution control (3.2×10^−12^)	0% (0/9)	>532
R6 PMCA product	100% (9/9)	243±8

### PrP^Sc^ distribution in the tissues of BSE-affected cattle

**Figure 6:**
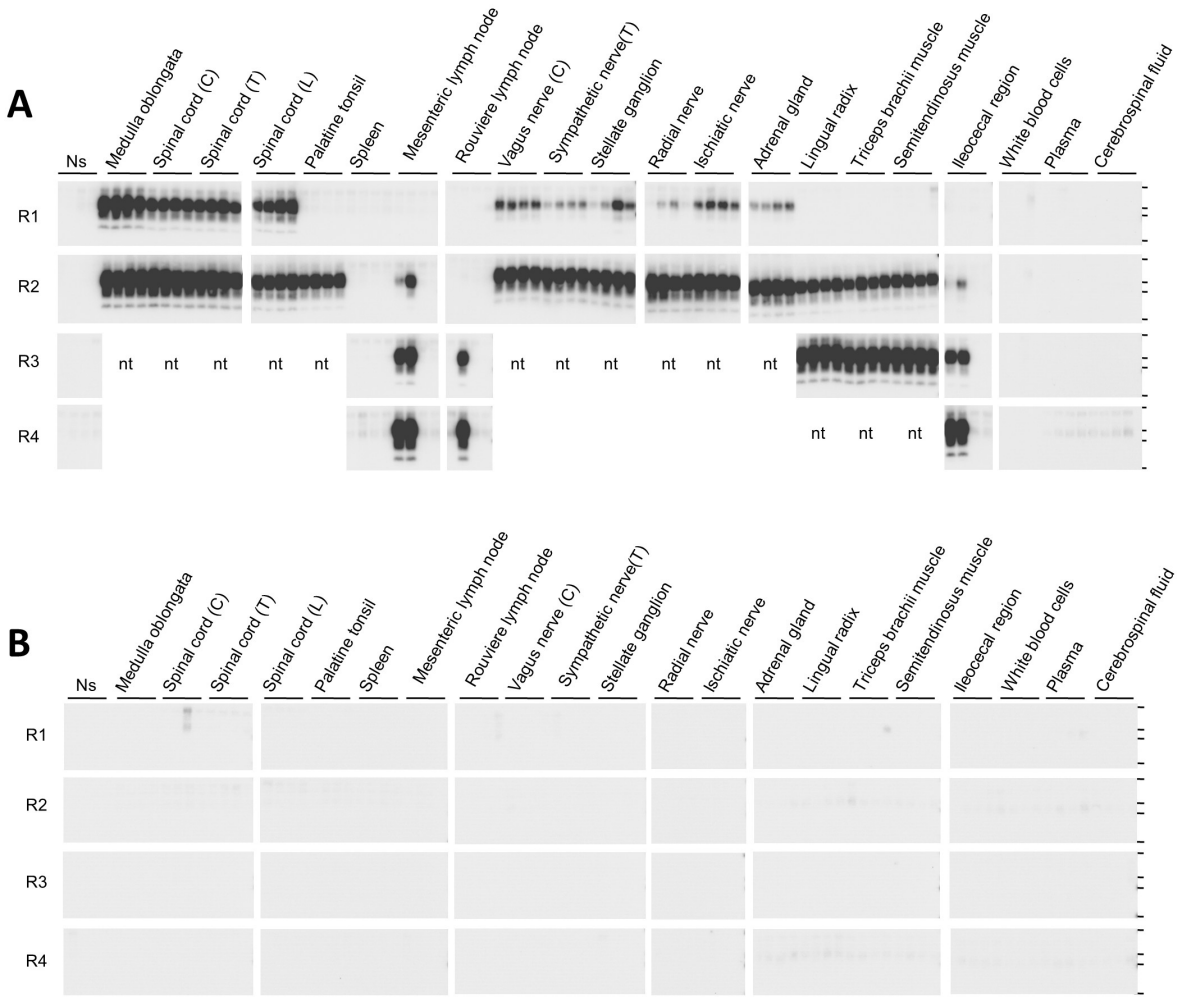
Distribution of PrP^Sc^ in cattle inoculated with BSE. **A**. Tissue distribution of PrP^Sc^ in the terminal disease stage in cow 5550. Quadruplicate samples of each tissue and bodily fluid were serially amplified, and the samples were analyzed after each round of amplification (R1–R4) by WB after digestion with PK. Horizontal lines indicate the positions of molecular weight markers corresponding to 37 kDa, 25 kDa, 20 kDa, and 15 kDa. Ns: No seed control. **B**. Negative control reaction for serial PMCA in each tissue. Quadruplicate samples of each tissue and bodily fluid from uninfected cow 2914 were serially amplified, and the samples were analyzed by WB following digestion with PK after each round of amplification. C: Cervical region, T: Thoracic region, L: Lumbar region, nt: Not tested.

Using our improved method, the distribution of PrP^Sc^ was examined in cattle that were orally administered a brain homogenate prepared from BSE-infected cattle. In the terminal disease stage in BSE-affected cow 5550, PrP^Sc^ was detected by conventional WB in several peripheral nervous tissues and the adrenal glands (Table 2). Moreover, PrP^Sc^ was detected by serial DSP-PMCA in the palatine tonsils, lymph nodes, ileocecal region, and muscular tissues (Figure 6A), whereas no PrP^Sc^ signal was detected in the corresponding tissue samples from uninfected control cow 2914 (Table 2 and Figure 6B).

**Table 2:** Detection of PrP^Sc^ by conventional WB analysis.

Cow Number	2914660	5499	5468	5550	9007
Months post administration	Control	34(p.o.)	42(p.o.)	57(p.o.)	17(i.c.)
Clinical status at dissection	Normal	Affected	Affected	Affected	Asymptomatic
Medulla oblongata	–	+	+	+	+
Spinal cord (cervical)	–	+	+	+	+
Spinal cord (thoracic)	–	+	+	+	+
Spinal cord (lumbar)	–	+	+	+	+
Palatine tonsil	–	–	–	–	ND
Spleen	–	–	–	–	ND
Mesenteric lymph node	–	–	–	–	ND
Rouviere lymph node	–	–	–	–	ND
Vagus nerve (cervical)	–	–	+	+	ND
Sympathetic nerve (thoracic)	–	–	–	–	ND
Stellate ganglion	–	+	+	+	ND
Radial nerve	–	–	–	–	ND
Ischiatic nerve	–	–	–	–	ND
Adrenal gland	–	–	+	+	ND
Lingual radix	–	–	–	–	ND
Triceps brachii muscle	–	–	–	–	ND
Semitendinosus muscle	–	–	–	–	ND
Ileocecal region	–	–	–	–	ND
White blood cells	–	–	–	–	–
Plasma	–	–	–	–	–
Cerebrospinal fluid	–	–	–	–	–

p.o.: peroral inoculation; i.c.: intracerebral inoculation. Negative symbols indicate that no PrP^Sc^ signal was detected in the samples before amplification. Positive symbols indicate that typical PrP^Sc^ signals were detected in the samples. ND: not determined.

**Figure 7:**
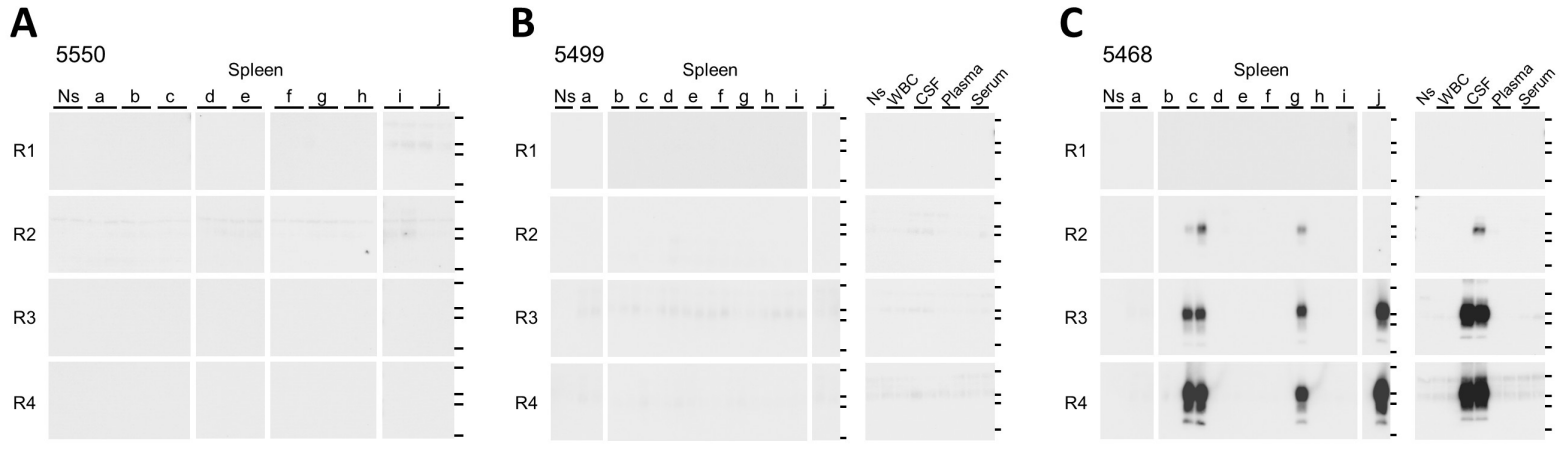
PrP^Sc^ in spleen and bodily fluid samples obtained in the terminal disease stage. A total of 10 tissue pieces (0.2 g each) were cut from the central portion of the spleen (a–e) and splenic hilum (f–j) for amplification. Duplicate samples of spleen tissue from BSE-affected cows 5550 (**A**), 5499 (**B**), and 5468 (**C**) were examined. Blood and CSF samples from cows 5499 (**B**) and 5468 (**C**) were also examined. Horizontal lines indicate the positions of molecular weight markers corresponding to 37 kDa, 25 kDa, 20 kDa, and 15 kDa. WBC: White blood cells, CSF: Cerebrospinal fluid, Ns: No seed control.

We could not detect PrP^Sc^ in the spleen, blood, or cerebrospinal fluid (CSF) from BSE-affected cow 5550 in the end stage of disease, even after four rounds of amplification. Although an additional five tissue pieces were cut from both the central portion of the spleen and the splenic hilum for amplification, splenic PrP^Sc^ was not detected in any of these 10 tissue samples (Figure 7A). The distribution of PrP^Sc^ in the spleen was examined further in two more terminal disease-stage BSE-affected cattle, numbers 5499 and 5468. As was the case with cow 5550, splenic PrP^Sc^ was not detected in any of the 10 tissue samples from cow 5499 (Figure 7B). However, a PrP^Sc^ signal was detected in 3 of 10 tissue pieces from the spleen of cow 5468 (Figure 7C).

### PrP^Sc^ distribution in the bodily fluids of BSE-affected cattle

Before amplification, PrP^Sc^ accumulation was confirmed using conventional WB analysis in the peripheral nervous tissues of cows 5499 and 5468 and in the adrenal gland of cow 5468 (Table 2). Individual differences in the distribution of PrP^Sc^ in CSF samples were observed in terminal-stage animals. PrP^Sc^ signals were detected in duplicate samples from cow 5468 after three rounds of amplification (Figure 7C) but were not detected in samples from cow 5499 (Figure 7B), as confirmed in samples from cow 5550 (Figure 6A).

**Figure 8:**
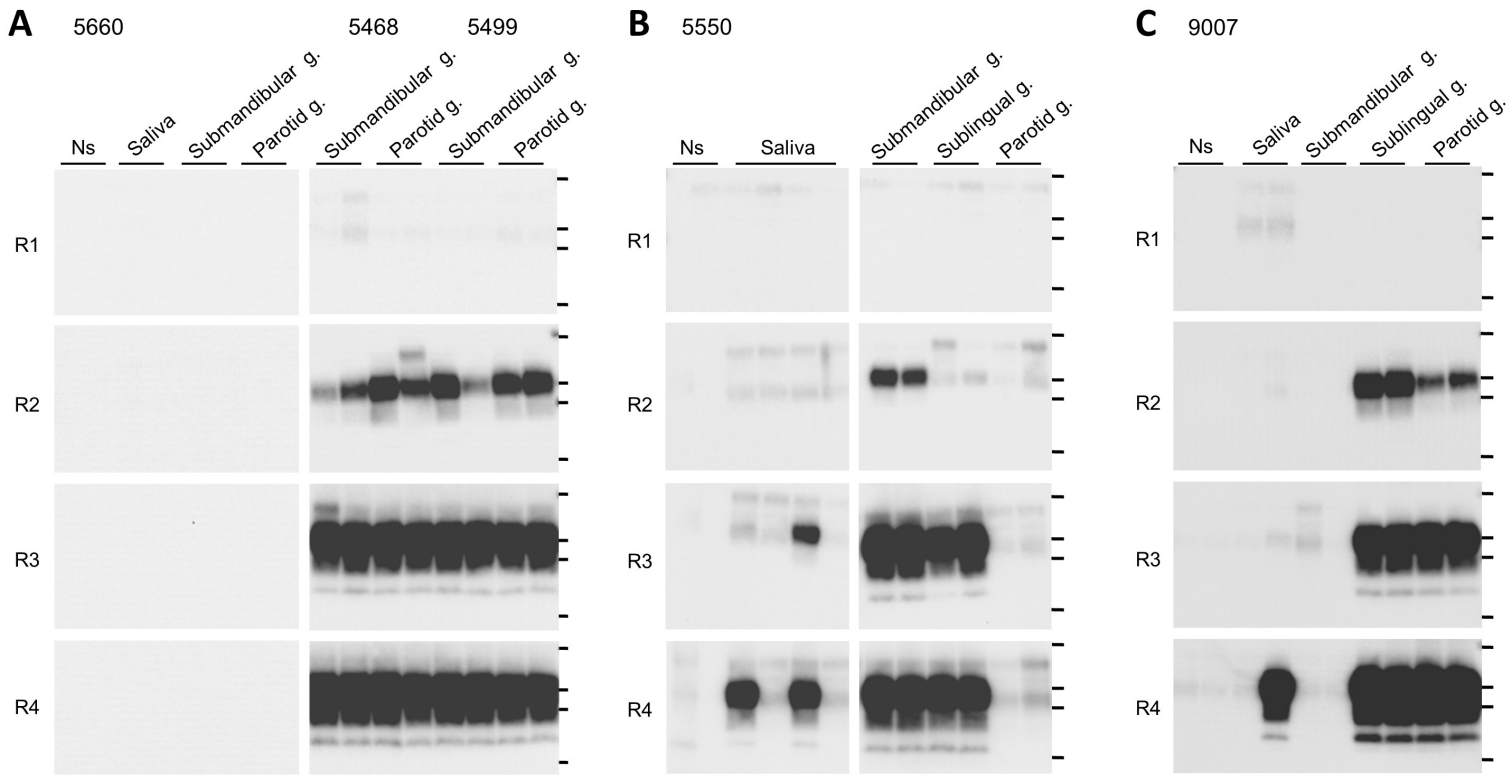
Detection of PrP^Sc^ by serial DSP-PMCA in saliva and salivary glands. Salivary PrP^Sc^ was evaluated in samples from an uninfected control cow (5660) and samples obtained from BSE-inoculated cows (5468, 5499, and 5550) in the terminal disease stage. Salivary PrP^Sc^ was also evaluated in an asymptomatic cow (9007) that had been intracerebrally inoculated with BSE. Horizontal lines indicate the positions of molecular weight markers corresponding to 37 kDa, 25 kDa, 20 kDa, and 15 kDa. Ns: No seed control.

We also examined the distribution of PrP^Sc^ in the salivary glands and saliva obtained from BSE-affected cattle. PrP^Sc^ was detected in the submandibular and parotid glands of cows 5468 and 5499 after two rounds of amplification (Figure 8A). In cow 5550, PrP^Sc^ was detected in the submandibular and sublingual glands (Figure 8B). Moreover, a PrP^Sc^ signal was detected in two of the quadruplicate saliva samples from cow 5550 after four rounds of amplification. The presence or absence of salivary PrP^Sc^ was investigated in intracerebrally infected cow 9007. PrP^Sc^ accumulation in the brain stem was found by conventional WB analysis 17 months after inoculation (Table 2), but the animal remained asymptomatic until sacrifice. PrP^Sc^ was detected in the sublingual and parotid glands after two rounds of amplification. In cow 9007, one of the duplicate saliva samples was found to be positive for PrP^Sc^ after four rounds of amplification (Figure 8C).

## Discussion

The present study is the first report describing ultrasensitive detection of BSE PrP^Sc^ in cattle. The determination that sulfated dextran compounds enhance the efficiency of *in vitro* BSE PrP^Sc^ amplification was unexpected because sulfated dextran has long been known as an antiscrapie agent both *in vivo* [26] and *in vitro* [27]. *In vitro* studies using recombinant PrP [28] and PrP^C^ purified from brain homogenate [29] have suggested that various polyanionic compounds increase the amplification of protease-resistant PrP. Therefore, endogenous polyanionic molecules such as sulfated glycans [28], [29] and RNA [29] could be cofactors required to facilitate the propagation of PrP^Sc^. Small and negatively charged dextran compounds (DSP and DSS-L) were most effective for *in vitro* amplification of bovine PrP^Sc^ in this study. These dextran compounds may accelerate the rate of PrP^Sc^ formation by acting as accessory molecules that facilitate or stabilize interactions between PrP^Sc^, PrP^C^ and cofactors contained in brain homogenates. Since species-specific differences in cofactor preference for *in vitro* amplification of PrP^Sc^ have been reported [30], dextran compounds may interfere with PrP^Sc^ formation by acting as competitive inhibitors of the cofactors required for propagation of scrapie PrP^Sc^.

The theory that PrP^Sc^ is widely distributed in various peripheral tissues in the terminal disease stage is strongly supported by our experimental results as well as previous findings, which demonstrated a low level of infectivity in the tonsils [31] and muscles [32] of cattle that were orally administered a brain homogenate prepared from BSE-infected cattle. With regard to the level of PrP^Sc^ detected in muscle tissue, the level estimated from the amplification factor in PMCA was lower than that found in a brain homogenate diluted to 10^−6^ because no PrP^Sc^ signal was detected in the first round of amplification. In the mesenteric and Rouviere lymph nodes as well as the ileocecal region, only one or two of the quadruplicate samples tested positive, even after four rounds of amplification. Given that PrP^Sc^ tends to aggregate, partial detection of PrP^Sc^ in the reaction may be due to the near absence of PrP^Sc^; PrP^Sc^ levels in these tissues would have been equivalent to the levels found in brain homogenate dilutions of 10^−10^ to 10^−11^.

Although significant prionemia [33] and PrP^Sc^ distribution in the blood [34] have been demonstrated in scrapie-infected animals, the presence of PrP^Sc^ in the blood was not substantiated in the BSE-affected cattle used in our study, despite a dramatic improvement in the sensitivity of PrP^Sc^ detection. This observation was in agreement with the previous result showing that the disease is not transmitted through the blood of BSE-affected cattle [32], [35]. Together with the scattered accumulation of PrP^Sc^ in the spleen and the low-level accumulation of PrP^Sc^ in the lymph nodes, the absence of detectable PrP^Sc^ in the blood of BSE-affected cattle suggests a neuronal rather than lymphoreticular progression of BSE PrP^Sc^ to the brain.

With regard to other bodily fluids, a bioassay of saliva from CWD-affected deer showed significant levels of infectious prions [36]. The presence of PrP^Sc^ in the salivary glands [37] and the amplification of PrP^Sc^ in concentrated buccal swab samples [38] have been reported in scrapie-infected sheep. Ours is the first report describing PrP^Sc^ detection in both the salivary glands and saliva of BSE-infected cattle. The salivary glands are regulated by parasympathetic nerves arising from the salivary nuclei of the medulla oblongata and sympathetic nerves arising from the thoracic portion of the spinal cord. Thus, it is possible that PrP^Sc^ accumulated in the central nervous system and spread to the salivary glands through the autonomic nervous system, and that very small amounts of PrP^Sc^ were secreted into the saliva in BSE-infected cattle.

Our study demonstrated that the distribution of PrP^Sc^ was not restricted to the nervous system, and that PrP^Sc^ was able to spread to most of the peripheral tissues examined in the terminal disease stage. The finding that PrP^Sc^ could be amplified in saliva taken from an asymptomatic animal suggests a potential usefulness of this technique for BSE diagnosis. Detailed examinations of the temporal course of BSE infection and the incidence of PrP^Sc^ appearance in saliva, as well as studies of how infection route affects salivary PrP^Sc^ accumulation, will be necessary to confirm whether salivary PrP^Sc^ can serve as a reliable marker for BSE infection. The highly sensitive method we describe has other practical applications as well, such as evaluating the safety of livestock products and raw feed materials, and safety assurance of pharmaceutical and cosmetic products manufactured from bovine source materials.

## Materials and Methods

### Ethics Statement

All animal experiments were approved by the Animal Care and Use Committee (approval IDs: 450 and 08-009) and Animal Ethics Committee (approval IDs: 04-III-7 and 08-IV-32) of the National Institute of Animal Health.

### Cattle

The cattle used in this study were imported from Australia. A c-BSE-infected brain homogenate (50 ml of a 10% homogenate) was orally administered to cattle (cows 5499, 5468, and 5550) ranging from 10 to 12 months in age. After 34 to 57 months, the animals were sacrificed and dissected in the terminal disease stage. Cow 9007 was intracerebrally inoculated with c-BSE-infected brain homogenate (1 ml of a 10% homogenate), sacrificed and dissected 17 months following inoculation, and the tissues were used for the analysis of salivary PrP^Sc^. Normal 3- to 4- month-old cattle (cows 2914 and 5660) were used as controls.

### Sample preparation

Tissues, white blood cells (WBCs), plasma, serum, CSF, and saliva were collected upon dissection and stored in small aliquots at −80°C. Oral cavity saliva was collected by aspiration. Samples from each tissue (0.2 g) and WBCs (approximately 2×10^8^ cells) were homogenized and suspended at 20% (w/v) in phosphate-buffered saline (PBS) containing 2× complete protease inhibitors (Roche Diagnostics).

### PrP^C^ substrate

To avoid contamination, brain homogenates were prepared in a laboratory that had never contained infected materials. The brains of bovine PrP^C^-overexpressing TgBoPrP transgenic mice and PrP knockout (PrP^0/0^) mice were homogenized separately in 20%(w/v) PBS containing 2× complete protease inhibitors. The homogenates were mixed with an equal volume of elution buffer (PBS containing 2% Triton X-100 and 8 mM EDTA) and incubated at 4°C for 1 h with continuous agitation. After centrifugation at 4500×*g* for 5 min, the supernatants were mixed in a 5∶1 proportion of PrP^0/0^:TgBoPrP. This mixture was used as the PrP^C^ substrate. Dextrans (Nacalai), dextran compounds (sodium dextran sulfate (DSS), potassium dextran sulfate (DSP), Nacalai; DEAE-dextran, Sigma), pentosan polysulfate (PPS, Elmiron, Janssen-Ortho), sulfated polysaccharide (λ-carrageenan (λ-Cag), Nacalai), glycosaminoglycans (sodium chondroitin sulfate C (CSS), sodium heparan sulfate (HSS), Nacalai), and heparan sulfate proteoglycan (HSPG, Sigma) were dissolved in PBS or distilled water and added to the PrP^C^ substrate at the concentration indicated in the figures. For the amplification of sheep scrapie PrP^Sc^, normal ICR mouse brains were homogenized in 10% (w/v) PBS containing complete protease inhibitors, 1% Triton X-100, and 4 mM EDTA. After centrifugation at 4500×*g* for 5 min, the supernatant was used as the PrP^C^ substrate.

### PMCA

To examine the bovine PrP^Sc^ detection sensitivity, 100 µl of PrP^C^ substrate containing 0.5% DSP was mixed with 1/100 volume of 10% brain homogenate from cattle infected with c-BSE (infectivity titer  = 10^6.7^ LD50/g) [20], and serial 10-fold dilutions were prepared of the PrP^C^ substrate containing 0.5% DSP. Homogenates of each tissue and WBCs, plasma, serum, CSF, and saliva were diluted 1∶20 with the PrP^C^ substrate containing 0.5% DSP (total volume 100 µl) in an electron beam-irradiated polystyrene tube. Amplification was performed with a fully automatic cross-ultrasonic protein-activating apparatus (Elestein 070-CPR, Elekon Science Corporation) using 40 cycles of sonication in which a 3-s pulse oscillation was repeated five times at 1-s intervals, followed by incubation at 37°C for 1 h with agitation. For the amplification of PrP^Sc^ in various tissues from BSE-inoculated and control cattle (Figure 6), the PrP^C^ substrate containing 0.5% DSP was mixed with 1/20 volume of homogenized samples or bodily fluids (total volume 80 µl) in an electron beam-irradiated 8-strip polystyrene tube specially designed for PrP^Sc^ propagation. PMCA was performed using 40 cycles of sonication (a pulse oscillation for 5 s, repeated five times at 1-s intervals), followed by incubation at 37°C for 1 h with agitation. The 1∶5 dilution of the PMCA product and subsequent amplification was repeated three times.

### Western blotting

Samples (10 µl) were mixed with 10 µl of proteinase K (PK) solution (100 µg/ml) after each round of amplification and incubated at 37°C for 1 h. The digested materials were mixed with 20 µl of 2× SDS sample buffer and incubated at 100°C for 5 min. The samples were separated by SDS-PAGE and transferred onto a polyvinylidene fluoride membrane (Millipore). After blocking, the membrane was incubated for 1 h with a horseradish peroxidase (HRP)-conjugated T2 monoclonal antibody [39] diluted 1∶10 000. After washing, the blotted membrane was developed using the Immobilon Western Chemiluminescent HRP Substrate (Millipore) according to the manufacturer's instructions. Chemiluminescence signals were analyzed with a Light Capture system (Atto).

### Bioassay

A 10% brain homogenate from c-BSE-infected cows was diluted to 10^−8^ with PrP^C^ substrate containing 0.5% DSP and amplified. The PMCA product was diluted 1∶5 with the PrP^C^ source containing 0.5% DSP, and a second round of amplification was performed. The 1∶5 dilution of the PMCA product and its subsequent amplification were repeated five times. The product from the sixth round was diluted 1∶10 with PBS and inoculated intracerebrally into TgBoPrP mice (20 µl per mouse). The PrP^C^ substrate containing 0.05% DSP and the PrP^C^ substrate containing the PrP^Sc^ seed diluted to 3.2×10^−12^ were inoculated as dilution controls for DSP and the PrP^Sc^ seed, respectively.

### Immunohistological analysis

The left hemisphere of the brain was fixed in 10% buffered formalin for neuropathological analysis. Coronal brain sections were immersed in 98% formic acid to reduce infectivity and embedded in paraffin wax. Sections of 4-µm thickness were cut and stained with hematoxylin and eosin (HE). Immunoreactive PrP^Sc^ was detected in brain sections using anti-PrP monoclonal antibody F99/97.6.1 (VMRD) or 12F10 (SPI-bio) as the primary antibody. An anti-mouse universal immunoperoxidase polymer (Histofine simple stain MAX-PO (M), Nichirei) was used as the secondary antibody, and 3,3′-diaminobenzidine tetrachloride served as the chromogen.
